# Determination of Ophthalmic Parameters and Ocular Morphology in Ring‐Necked Parakeets (*Psittacula krameri*)

**DOI:** 10.1111/vop.70032

**Published:** 2025-07-01

**Authors:** Fernanda Taques Wendt, Fabiano Montiani‐Ferreira, Cecília Capacchi Dall’agnol, Franz Riegler Mello, Thiago Francisco Costa Solak, Breno Castello‐Branco Beirão, Marcello Machado, Rogério Ribas Lange

**Affiliations:** ^1^ Department of Veterinary Medicine Federal University of Paraná (UFPR) Brazil; ^2^ Department of Basic Pathology, Laboratory of Compared Immunology Federal University of Paraná (UFPR) Brazil; ^3^ Department of Anatomy Federal University of Paraná (UFPR) Brazil

**Keywords:** endodontic absorbent paper points, esthesiometry, intraocular pressure, ocular ultrasonography, *Psittacula krameri*, ring‐necked parakeet

## Abstract

**Purpose:**

To determine normal ophthalmic test values and to describe the ocular morphology of ring‐necked parakeets ( *Psittacula krameri* ).

**Materials and Methods:**

33 captive birds were examined, although not all tests were conducted on each bird. The tests included endodontic absorbent paper point tear test (EAPPTT), intraocular pressure (IOP), corneal touch threshold (CTT), central corneal thickness (CCT), palpebral and corneal diameters, ocular ultrasonography, and corneoconjunctival microbiota. The iris coloration of seven young parakeets was evaluated, and two macerated skulls were described.

**Results:**

Normal values obtained were: EAPPTT: 7.57 ± 1.95 mm/min; IOP: 12.47 ± 1.51 mmHg; CTT: 2.46 ± 0.5 mm; CCT: 0.12 ± 0.006 mm; palpebral diameter: 8.04 ± 0.49 mm,; corneal diameter: 6.41 ± 0.49 mm. In ocular ultrasonography, the dimensions in the sagittal plane were: axial globe length (AGL): 0.94 ± 0.02 cm; vitreous chamber depth (VCD): 0.47 ± 0.02 cm; lens axial length (LAL): 0.32 ± 0.01 cm; anterior chamber depth (ACD): 0.1 ± 0.01 cm; pecten width (PW): 0.12 ± 0.02 cm, and pecten length (PL): 0.42 ± 0.04 cm. In the dorsal plane: AGL: 0.93 ± 0.03 cm; VCD: 0.47 ± 0.02 cm; LAL: 0.32 ± 0.03 cm; ACD: 0.1 ± 0.01 cm; PW: 0.32 ± 0.04 cm, and PL: 0.35 ± 0.03 cm. Microbiota analysis identified predominantly gram‐positive bacteria, with *Staphylococcus* sp. being the most common (47.5%). Anatomical evaluation of skulls revealed a large lacrimal process extending to the zygomatic process, and the margins of the processes intersect but do not fuse. Young parakeets exhibit a brownish iris that gradually turns blue by 60 days of age.

**Conclusion:**

These findings may contribute to the diagnosis of ophthalmic conditions in this species and provide insight into normal anatomical features.

## 1 Introduction

The Order Psittaciformes includes approximately 400 species, many of which are popular pets worldwide. The ring‐necked parakeet ( *Psittacula krameri* ) belongs to the genus *Psittacula*. It is a medium‐sized bird, approximately 40 cm long, and a distinctive feature of this species is its large, curved, reddish‐colored upper beak. Males of the species have a black ring around their neck, indicating sexual maturity, which is reached at around three years old [1, 2]. These birds attract attention from breeders because of their variety of color mutations and their intelligence in learning tricks and sounds, which makes them highly interactive. This species is native to India and sub‐Saharan Africa, and according to the IUCN [3], its conservation status is of least concern. The ring‐necked parakeet is recognized as an invasive species due to its adaptability to different ecological niches and generalist diet, in addition to its popularity as a pet. This has made it the most abundant psittacine in Europe, despite being an exotic species. The establishment of these populations has both direct and indirect impacts on native wildlife and humans, including economic losses due to interactions with crops [2, 4].

A complete ophthalmic examination is essential to determine when an abnormality is present. Additionally, species‐specific parameters are necessary, as there are anatomical and functional variations among the eyes of birds [5] due to differences in their ecology, which influence both eye adaptations and visual behaviors [6]. Ophthalmic parameters have been described for various psittacine species, including macaws [7, 8], parrots [9, 10, 11], cockatiels [12], quaker parakeets [13], and budgerigars [14]. Regarding studies on ring‐necked parakeets, one study determined intraocular pressure values [15]. Therefore, further research is important to gather additional data on the ophthalmology of this species, which is commonly seen in clinical avian practice.

The aim of this study is to characterize the eyes and bony orbit of the ring‐necked parakeet, describing aspects of its anatomy, corneal thickness, and ocular biometry measurements. Additionally, it seeks to obtain diagnostic values for selected ophthalmic tests related to tear production, intraocular pressure, corneal sensitivity, and ocular microbiota. This information will contribute to reference values for diagnostic tests used in assessing the ocular health of ring‐necked parakeets, aiding in the accurate identification of ophthalmic diseases affecting this species.

## 2 Materials and Methods

All procedures involving live birds were performed after receiving approval from the Animal Ethics Committee of the Federal University of Paraná (UFPR) and based on the “Guidelines for the Use of Animals in Ophthalmic Research” by the Association for Research in Vision and Ophthalmology (ARVO). These birds were part of the breeding stock at Exotic Animal Incubation and Breeding Laboratory, located in Experimental Farm Canguiri of the Federal University of Paraná (UFPR), Pinhais, PR, Brazil. The study was designed with a sample size of 33 adult ring‐necked parakeets (15 females and 18 males). A power analysis using Cohen's d for an independent samples *t*‐test was performed with the *TTestIndPower* function, Python version 3.8.10 (Python.org. https://www.python.org) to calculate the required sample size for a two‐tailed *t*‐test, based on the observed standard deviation, α = 0,05 (significance level 5%), and power = 0,80 (80% probability of detecting a true effect) [16]. All measurements exceeded the required sample sizes, and parametric tests were used for group comparisons. Not all ophthalmic tests were conducted on every bird due to transportation limitations and equipment availability.

For the morphological study of the bony orbit, two heads of birds that died of natural causes were used, and no animal was sacrificed for this research. The selection of these animals was justified by their routine inclusion in ongoing health monitoring as part of the aviary's standard management protocols. The birds were not captured or used solely for research purposes, but were already part of an established breeding population. The ophthalmic tests integrated into their regular check‐ups allowed for non‐invasive health evaluations, contributing to the birds' overall well‐being and allowing for early detection of potential eye conditions.

For observations of external ocular morphology and variations in iris coloration, eight chicks of undetermined sex and varying ages, born at Exotic Animal Incubation and Breeding Laboratory, were evaluated. No ophthalmic tests were performed on these chicks; only growth monitoring and photographic records were made at three time points: on the first day, after 20 days, and after 106 days.

### 2.1 Physical and Ophthalmic Evaluation

Before performing the selected ophthalmic tests, the birds underwent a physical examination, along with blood collection for a hematological and biochemical profile to rule out potential systemic conditions.

The birds were physically restrained by experienced assistants using towels. Each bird was held in an upright position with its head, wings, and pelvic limbs stabilized to ensure the safety of both the animal and the handler. The assistant's fingers were positioned along the sides of the bird's face, taking care not to apply pressure to the neck, allowing for secure control of the head and the beak. The wings were folded against the bird's body inside the towel to restrict movement and prevent injury, while the pelvic limbs were firmly held.

To minimize potential bias, a structured methodology was implemented when performing ophthalmic tests. The birds were individually identified, housed in separate enclosures during testing, and examined sequentially to maintain consistency in data collection. Each bird was weighed on a digital scale and underwent a physical examination, which included assessing body condition score, heart and respiratory auscultation, feather quality, the presence of ectoparasites and visible lesions. The ocular surface was examined by slit‐lamp biomicroscopy, and the selected birds showed no clinical or ophthalmic problems.

### 2.2 Ophthalmic Tests

**FIGURE 1:**
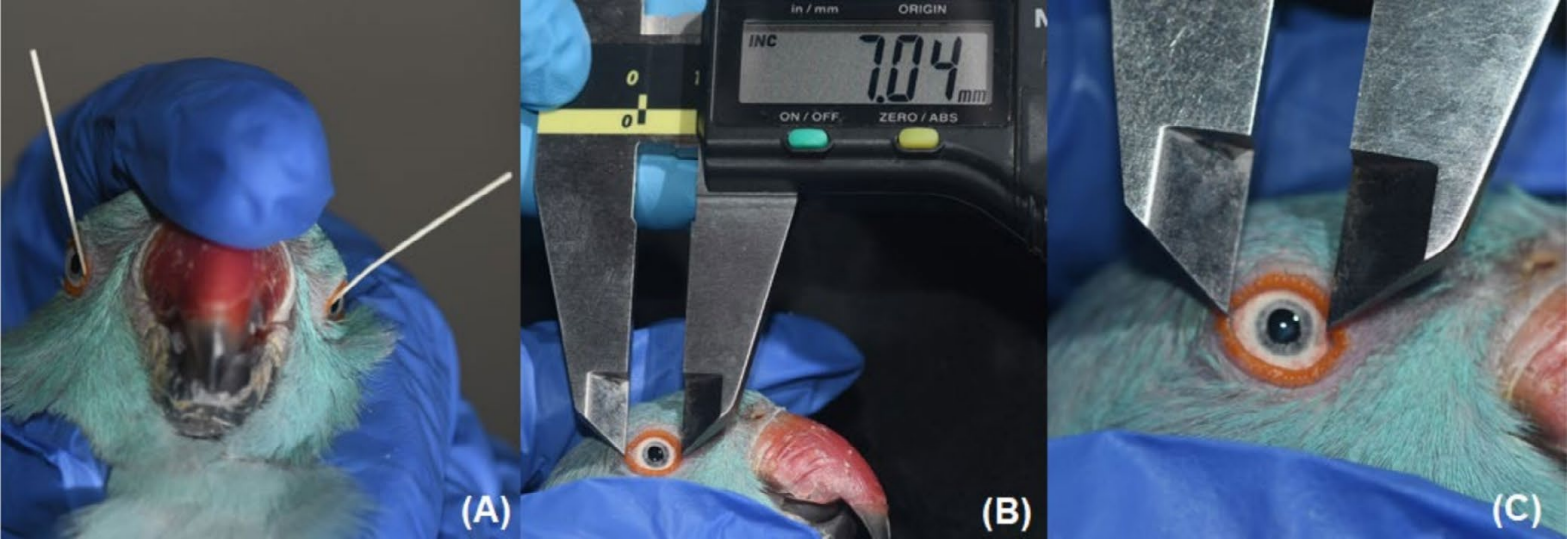
Ophthalmic tests are being performed on ring‐necked parakeets ( *Psittacula krameri* ) in captivity. (A) Endodontic Absorbent Paper Point Tear Test (EAPPTT); (B) Palpebral fissure length measurement; (C) Corneal diameter measurement.

**FIGURE 2:**
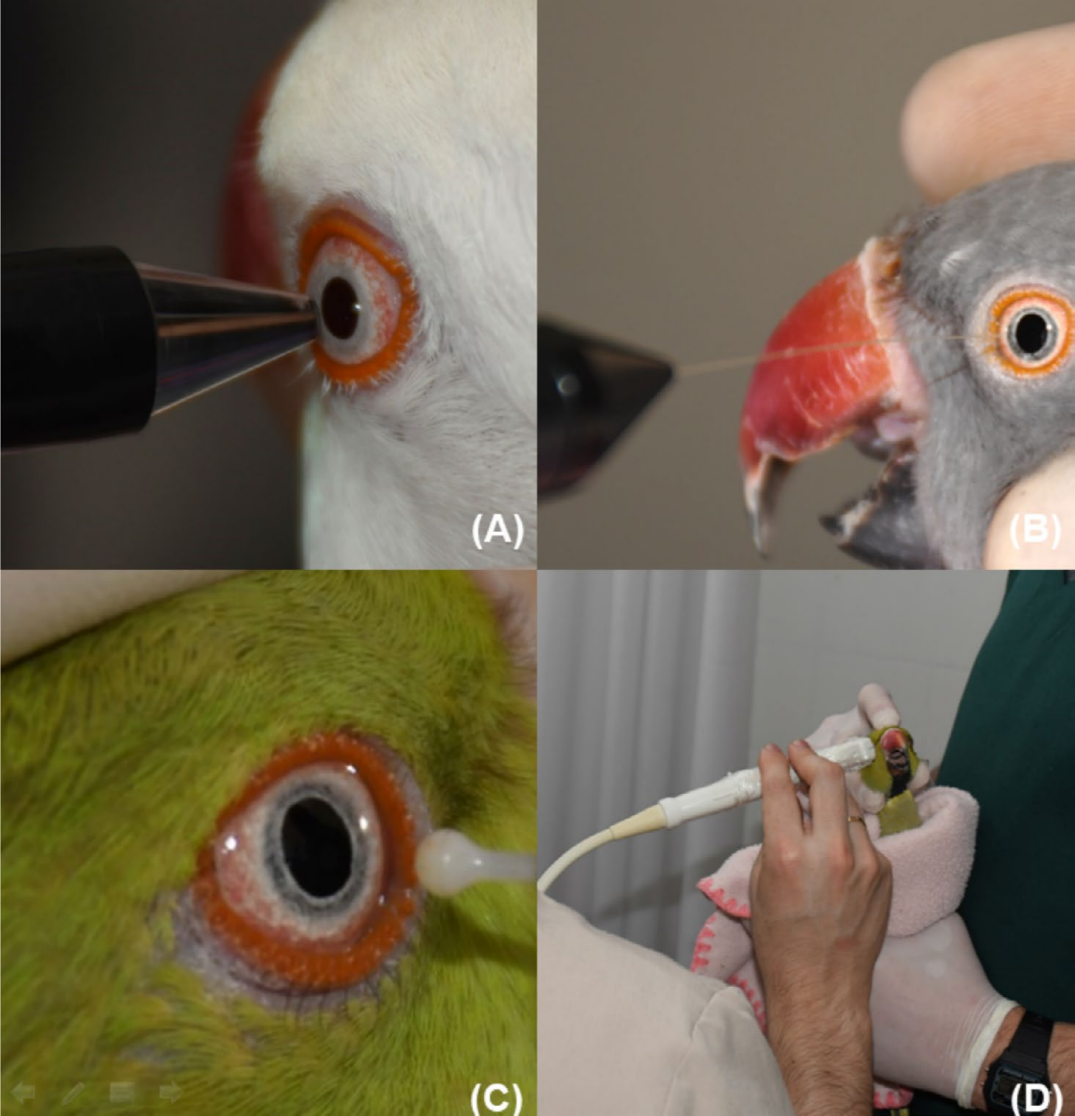
Ophthalmic tests are being performed on ring‐necked parakeets ( *Psittacula krameri* ) in captivity. (A) Central Corneal Thickness (CCT); (B) Corneal Touch Threshold (CTT); (C) Intraocular Pressure (IOP); (D) B‐mode scan ocular ultrasonography.

The ophthalmic tests (Figure 1 and Figure 2) were performed on the birds using manual restraint, and were conducted in three phases. In the first phase, the following tests were conducted: endodontic absorbent paper point tear test (EAPPTT) with endodontic paper points (Dentsply, Color size 30), intraocular pressure (IOP) using a rebound tonometer (Tonovet Plus, Icare Finland Oy), and measurement of the corneal and palpebral fissure diameters with a digital caliper (Mitutoyo). A month later, in the second phase, corneal–conjunctival swab samples were collected using Stuart and Amies charcoal transport medium. In the third phase, corneal touch threshold (CTT) was performed using a Cochet‐Bonnet esthesiometer (Cochet‐Bonnet, Luneau Ophtalmologie), central corneal thickness (CCT) was measured with an ultrasonic pachymeter (Micropach Sonomed, Model 200P+, Lake Success), and ocular biometry was conducted using B‐mode ultrasonography. For parameters measured in both eyes, the order of eye examination was alternated between birds to prevent measurement bias.

For the EAPPTT (Figure 1A), in 64 eyes from 32 animals, an endodontic paper point was inserted into the conjunctival sac and left in place for 60 s. This process was conducted simultaneously on both eyes of the birds under physical restraint. After the time elapsed, the paper point was removed, and the wet portion was immediately observed and measured in millimeters using a digital caliper. The average temperature recorded was 11°C, and the humidity was 96.8%.

After measuring tear production, the IOP was performed using a rebound tonometer in Canine mode (Figure 2C). The device was positioned vertically, perpendicular to the center of the cornea. The recorded IOP measured was averaged across three separate readings of six successive measurements from both the right and left eyes of 33 birds. Only results with a deviation of ≤ 1.8 mmHg were considered.

For the measurements of palpebral fissure length and corneal diameter, the birds were placed in lateral recumbency, and the measurements were taken using a digital caliper on both the right and left eyes of 33 birds. To measure the horizontal corneal diameter (Figure 1C), the caliper tips were positioned at the corneal limbus. For the palpebral fissure length (Figure 1B), the caliper tips were placed on the lateral and medial canthi of the eyelids. The average time to perform these four tests was six minutes per bird.

In the second phase of the project, material collection for the evaluation of corneal‐conjunctival microbiota was performed by gently swabbing the lower conjunctival fornix and ocular surface. To avoid sample contamination, care was taken not to touch the eyelid margin. Samples were collected from 34 eyes, and swabs were collected using Amies charcoal transport medium and Stuart transport medium. The samples were sent to the Polytechnic Center's Pathology Laboratory at the Federal University of Paraná for bacterial identification. The swabs were streaked on Petri dishes with sheep blood agar and incubated at 37°C for 24 h. In cases where bacterial growth was observed, subcultures were made on MacConkey and Tryptic Soy Agar (TSA) medium. For anaerobic microbiota determination, the swab samples were also cultured in thioglycolate medium and incubated under anaerobic conditions at 37°C for 24 h. Fungal microbiota was assessed by culturing the samples on Sabouraud agar, incubated at 27°C for seven days. After microbial growth, the samples underwent identification based on routine laboratory procedures, evaluating morphological and biochemical characteristics to classify the microorganisms.

In the third phase of the project, CTT was evaluated using a Cochet‐Bonnet esthesiometer (Figure 2B) on 28 eyes from 14 animals. While the birds were under physical restraint, the central corneal surface was touched with the nylon filament of the device, progressively shortening the filament until an eyelid movement was observed. CCT was measured in 28 eyes of 14 animals using an ultrasonic pachymeter (Figure 2A). The pre‐established ultrasonic sound speed in the equipment was 1640 m/s. The measurements were performed with birds under physical restraint, positioning the probe tip perpendicular to the cornea at the center of the pupil. Three measurements were taken for each eye, and the average was recorded.

**FIGURE 3:**
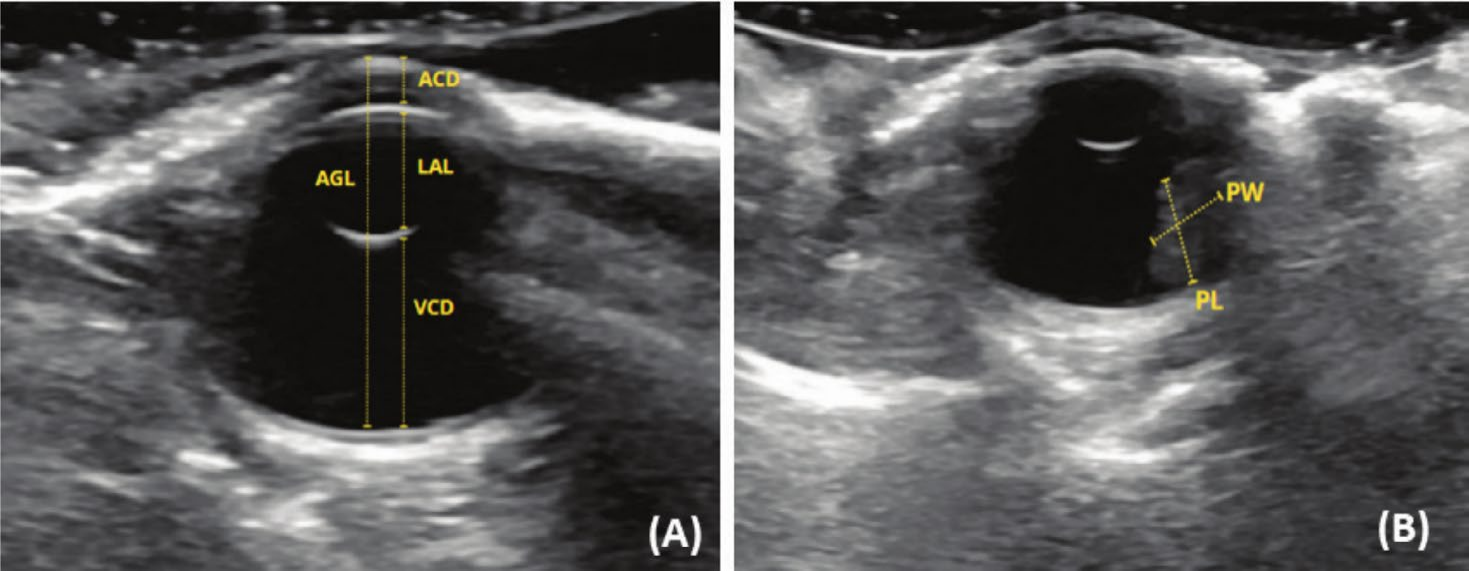
B‐mode ultrasound image of a healthy ring‐necked parakeet *(Psittacula krameri*) eye. (A) Sagittal plane. ACD, Anterior chamber depth; AGL, Axial length of the eyeball; LAL, Axial length of the lens; VCD, Vitreous chamber depth. (B) Dorsal plane showing the pecten. PL, Pecten length; PW, Pecten width.

Ocular biometry measurements were performed using ultrasonography in 28 eyes of 14 birds using B‐mode ultrasound with a 14‐MHz probe (Figure 2D). The exam was performed with the birds under physical restraint, with a drop of topical anesthetic eye drops based on proxymetacaine (Anestalcon) instilled into the eyes. The probe was positioned on the eyeball using gel for ultrasonography evaluation. Biometry was performed in the sagittal and dorsal sections of both eyes. The sagittal plane (Figure 3A) was oriented longitudinally to the body axis, while the dorsal plane (Figure 3B) divided the eye into dorsal and ventral segments. The following points were measured: axial globe length (AGL), vitreous chamber depth (VCD), axial length of the lens (LAL), anterior chamber depth (ACD), width and length of the ocular pecten (PW and PL). The images were taken by the same evaluator, ensuring the results were not influenced by differences in technique.

### 2.3 Preparation of the Dried Skulls for Anatomical Analysis

The heads of two adults of undetermined age, one male and one female, were used. Both died of natural causes at UFPR Exotic Animal Incubation and Breeding Laboratory. A combination of two maceration techniques was used to prepare the head bones: fly larvae and immersion in water. After removing the skin and eyeballs, the head was placed in a container covered by a perforated screen, which allowed access by flies from the environment. The larvae that hatched from the eggs consumed most of the soft tissue. After a week, the head was immersed in a container with water for another two weeks to complete the removal of the remaining tissue debris. To whiten the skull, it was immersed in a 50% hydrogen peroxide solution for 24 h, followed by washing in running water and drying at room temperature. The skull was then assembled with cyanoacrylate adhesive, keeping the mandible separated. The eyeballs were carefully dissected to remove the bulbar conjunctiva and extraocular muscles. After a cruciform incision of the cornea with the aid of a scalpel, they were washed in running water to remove internal components with the aid of a cotton swab, in order to preserve only the cartilaginous lamina of the sclera and the ring of scleral ossicles. After cleaning, the eyeballs were placed to dry at room temperature, maintaining their anatomical shape. The remaining tissues on the ring of scleral ossicles were removed with the aid of anatomical forceps and scraping with a scalpel. The anatomical study was performed with the naked eye, and the parts of interest were digitally photographed. The anatomical nomenclature used follows the *Nomina Anatomica Veterinaria* and the *Nomina Anatomica Avium*.

### 2.4 Statistical Analysis

A Shapiro–Wilk test was used to assess normality. The descriptive statistical analysis and presentation of data were performed using the mean value ± standard deviation. The Student's *t*‐test was used to compare the mean weight of males and females, and all ophthalmic parameters between the right and left eyes and between males and females, with a significance level of *p* < 0.05. Descriptive and inferential statistical analyses were all performed using MedCalc Statistical Software version 20.027 (MedCalc Software Ltd., Ostend, Belgium) and *Python 3.8.10* (Python.org. https://www.python.org).

## 3 Results

### 3.1 Physical Examination

Thirty‐three adult ring‐necked parakeets of unknown age were evaluated: 18 males (54.5%) and 15 females (45.5%). The average weight of males was 0.128 kg (±7.6) and that of females was 0.130 kg (±12.3). There was no significant difference (*p* < 0.05) in the average weight between the sexes. On physical examination, the birds were in good general condition. In order to evaluate changes in iris coloration in young individuals, eight individuals aged ranging from 13 days to six months were examined.

### 3.2 External Morphological Observations in Adults and Chicks

**FIGURE 4:**
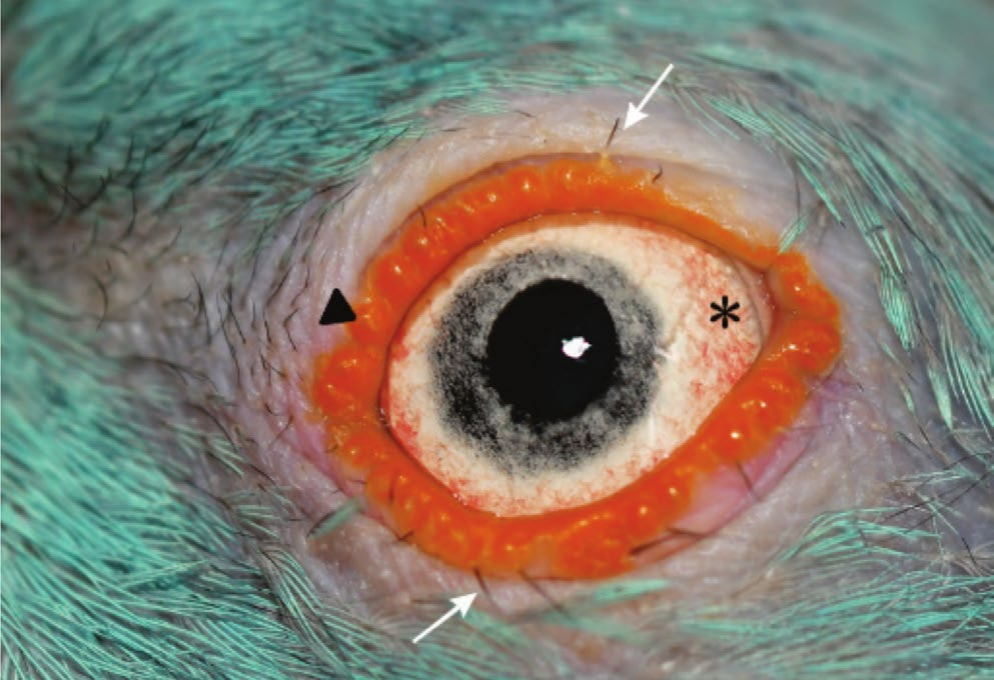
Healthy left eye of an adult female ring‐necked parakeet ( *Psittacula krameri* ). Note an apteric area around the eyes. The eyelids have rounded orange papillae (arrowhead) and black filaments scattered around the upper and lower eyelids (white arrows). The sclera is white and surrounded by reddish vasculature (asterisk). The iris is bluish in color, with black and white dots giving it a granular appearance, and the pupil is rounded.

The eyes of ring‐necked parakeets are positioned on the sides of the head, a characteristic similar to that of other birds of the order Psittaciformes. The eyelids are orange, and their anterior surface is covered by skin with rounded papillary projections (Figure 4). They have a few short, black phylloplumes on the upper and lower margins, without uniform distribution. There are no feathers on the eyelid margins, delimiting a circular apteric area that extends to the nostril.

**FIGURE 5:**
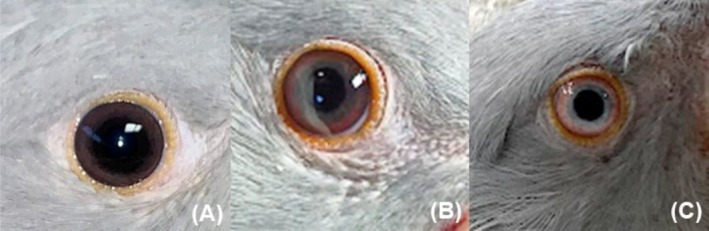
Chronology of iris color change in a juvenile ring‐necked parakeet ( *Psittacula krameri* ) of indeterminate sex. (A) At 42 days, the iris is brownish in color, and the sclera is barely visible. (B) At 63 days, the iris gradually changes color to a lighter brownish tone, and the sclera is barely visible. (C) At 168 days, the iris is bluish in color, and the sclera is visible with peripheral blood vessels, similar to that observed in an adult individual.

The third eyelid is located at the medial angle of the eye. It is moderately opaque, poorly pigmented, and has apparent vascularization. The pupil is circular and exhibits very evident and rapid voluntary control. The iris is light blue, with whitish and black dots, giving a grainy appearance in adults. Depending on the mutation in the animal's feather color, the iris takes on different shades of blue, which may be grayish or lighter. No sexual dimorphism was observed in relation to iris color. The sclera has peripheral vascularization, which gives it a pinkish color. In juveniles, changes in iris color were observed according to age chronology (Figure 5). On average, up to 60 days of age, the iris has a brownish coloration, and there is little visibility of the sclera's edge. From approximately 100 days of age, there is a gradual change, and the color appears closer to that of an adult, with bluish tones and peripheral vascularization delimitation in the sclera. Variations may occur depending on the plumage color mutation. In juveniles with the lutin mutation, the iris is reddish instead of brown and gradually acquires a bluish hue.

### 3.3 Ophthalmic Tests

The results obtained in the ophthalmic tests performed on captive adult ring‐necked parakeets selected for the study are shown in Table 1. There was no significant difference (*p* < 0.05) assessed by the Student's *T*‐test between the measurements in the right and left eyes of all the ophthalmic tests evaluated. Only in the evaluation of the CCT, there was a difference (*p* = 0.026) between males (mean 0.11 mm ± 0.006) and females (mean 0.12 mm ± 0.009), with seven individuals of each sex being evaluated.

**TABLE 1:** Values obtained for ophthalmic diagnostic tests in ring‐necked parakeets ( *Psittacula krameri* ), with the number of eyes evaluated (*N*) and statistical values of mean, standard deviation (SD), and median.

Ophthalmic test	*N*	Mean	SD	Median
EAPPTT (mm/min)	64	7.57	±1.95	7
IOP (mmHg)	66	12.47	±1.51	12
Palpebral fissure lenght (mm)	66	8.04	±0.49	8.08
Corneal diameter (mm)	66	6.41	±0.39	6.49
CTT (cm)	28	2.46	±0.5	2.25
CCT (mm)	28	0.12	±0.006	0.11

Abbreviations: CCT, central corneal thickness; CTT, corneal touch threshold; EAPPTT, endodontic absorbent paper point tear test; IOP, intraocular pressure; mm, millimeters; mm/min, millimeters per minute; mmHg, millimeters of mercury.

### 3.4 Analysis of Ocular Microbiota

Of the 34 samples collected, there was aerobic bacterial growth in 21 (61.76%), and 13 samples (38.23%) were negative. There was no growth of anaerobic bacteria or fungi. Table 2 presents the number of isolated bacteria and their frequency, dividing them into gram‐positive and gram‐negative.

**TABLE 2:** Results of the analysis of the ocular microbiota of ring‐necked parakeets ( *Psittacula krameri* ) in captivity.

Bacterias	*N*	Frequency
**Presence of bacterial growth**	21	
Gram‐positives	12	57.14%
*Staphylococcus* sp	5	23.8%
*Staphylococcus aureus*	3	14.28%
*Staphylococcus epidermidis*	2	9.52%
*Stomatococcus* sp.	1	4.76%
*Listeria* sp.	1	4.76%
Gram‐negatives	9	42.85%
*Klebsiella* sp.	3	14.28%
*Neisseria* sp.	2	9.52%
*Enterobacter aglomerans*	1	4.76%
*Moraxella* sp.	1	4.76%
*Pseudomonas mallei*	1	4.76%
*Yersinia* sp.	1	4.76%

The majority of bacterial isolates were gram‐positive cocci bacteria, representing 52.38% (11/21) of the total samples with positive bacterial growth, followed by gram‐negative bacilli with 33.3% (7/21). Gram‐negative cocci, coccobacilli, and gram‐positive bacilli had growth of only one bacterial isolate each. The most frequently isolated bacteria were from the genus *Staphylococcus* spp., with 47.61% (10/21), followed by *Klebsiella* sp. (14.28%, 3/21) and *Neisseria* sp. (9.52%, 2/21). In one sample each, *Yersinia* sp., *Stomatococcus* sp., *Moraxella* sp., *Listeria* sp., *Pseudomonas mallei* , and *Enterobacter agglomerans* were found.

### 3.5 Ocular Ultrasonography

All 28 eyes evaluated had a similar appearance on ultrasonographic examination. The cornea was hyperechoic and moderately curved, and the lens was visualized as two hyperechoic curved lines. The anterior and vitreous chambers are anechoic. The ocular pecten is hyperechoic and visible in the vitreous chamber as a tubular structure that protrudes from the retina. The results of mean values and standard deviation (in centimeters) of the ultrasonographic measurements performed in the sagittal and dorsal planes are presented in Table 3. There was no statistically significant difference between the right and left eyes.

**TABLE 3:** Results of B‐mode ultrasound measurements in the sagittal and dorsal planes, in centimeters, of 28 eyes of adult ring‐necked parakeets ( *Psittacula krameri* ) in captivity.

Ultrasound measurement	Mean S ± SD	Mean D ± SD
AGL	0.94 ± 0.02	0.93 ± 0.03
VCD	0.47 ± 0.02	0.47 ± 0.02
LAL	0.32 ± 0.01	0.32 ± 0.03
ACD	0.1 ± 0.01	0.1 ± 0.01
PW	0.12 ± 0.02	0.32 ± 0.04
PL	0.42 ± 0.04	0.35 ± 0.03

Abbreviations: ACD, anterior chamber depth; AGL, axial length of the globe; D, dorsal plane; PL, pecten length; PW, pecten width; S, sagittal plane; SD, standard deviation; VCD, vitreous chamber depth.

### 3.6 Anatomical Observations on the Macerated Skull

The ring of scleral ossicles (Figure 7B) has a circular shape, containing thirteen bony plates in 4 eyes analyzed, which have a trapezoidal shape and overlap each other to form a complete circle.

**FIGURE 6:**
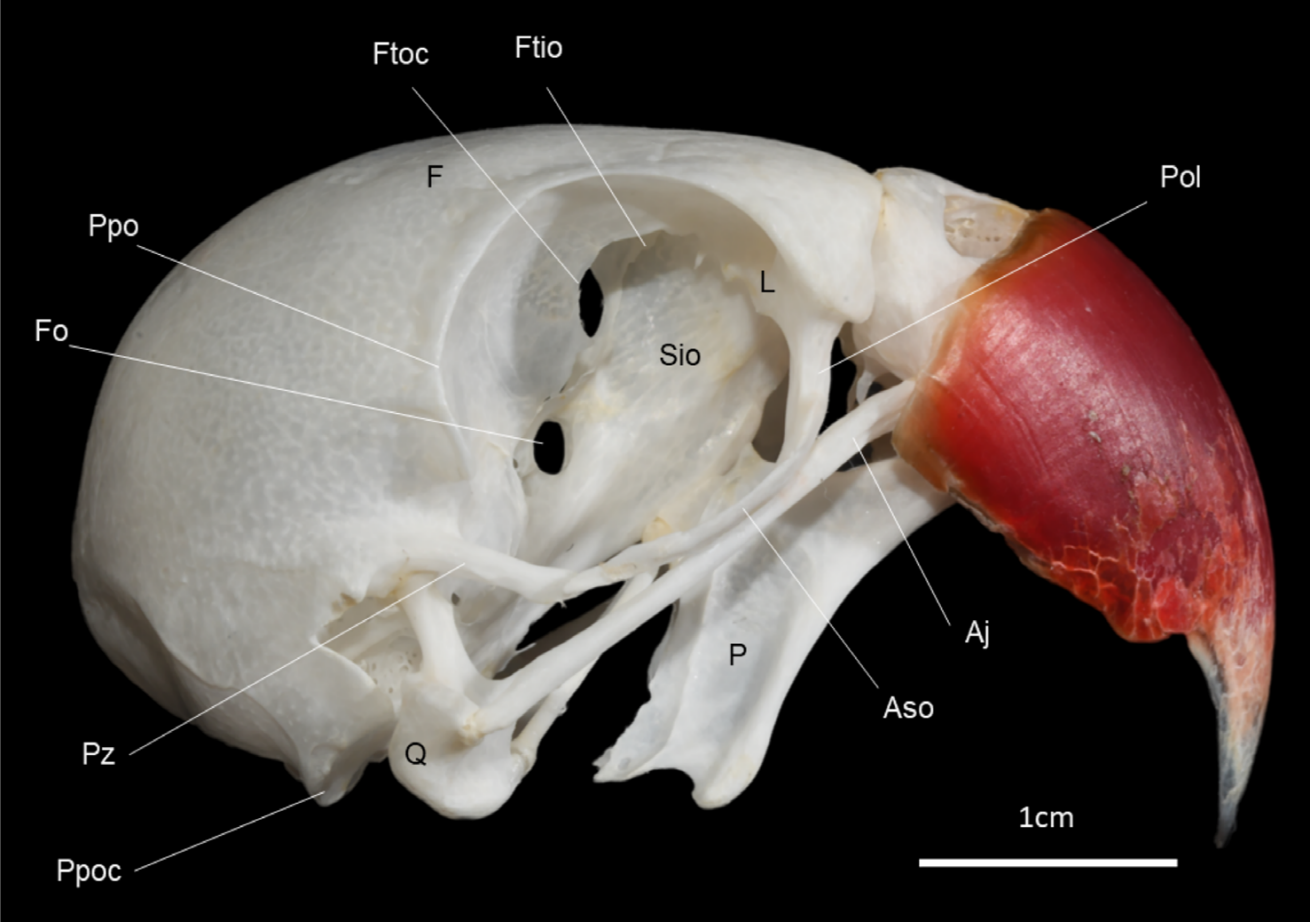
Lateral view of the skull (without mandible) of an adult male ring‐necked parakeet ( *Psittacula krameri* ). Aj, Jugal arch; Aso, Suborbital arch; F, Frontal bone; Fo, Optic foramen; Ftio, Interorbital fontanelle; Ftoc, Orbitocranial fontanelle; L, Lacrimal bone; P, Palatine bone; Pol, Orbital process of the lacrimal bone; Ppo, Postorbital process; Ppoc, Paraoccipital process; Pz, Zygomatic process; Q, Quadrate bone; Sio, Interorbital septum.

**FIGURE 7:**
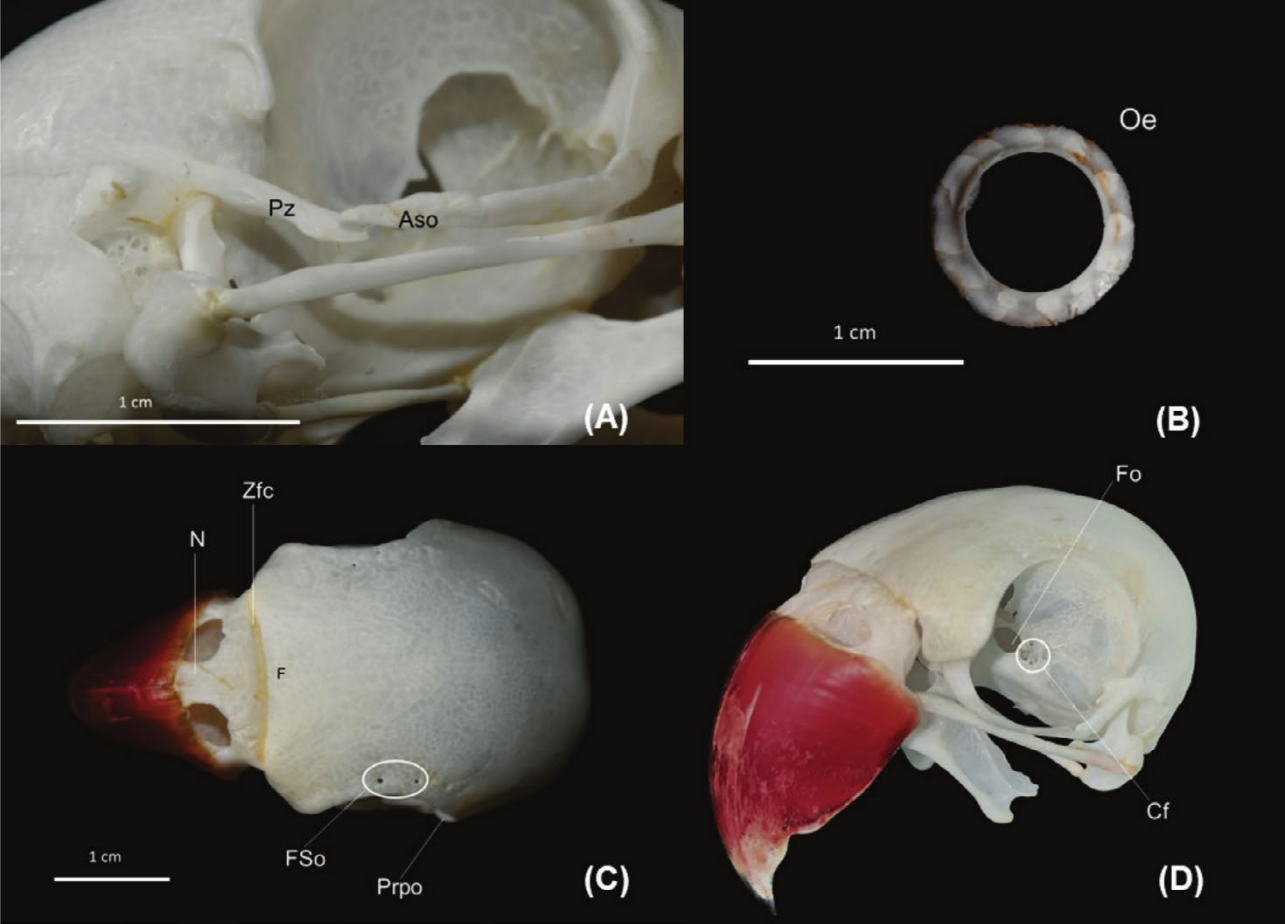
Skull and components of the fibrous tunic of the eyeball of an adult ring‐necked parakeet *(Psittacula krameri* ). (A) Enlargement of the orbital region in the ventrolateral aspect, evidencing the proximity of the suborbital arch and the zygomatic process, which cross but do not fuse; (B) Lateral aspect of the ring of scleral ossicles; (C) Dorsal aspect of the skull; (D) Rostrolateral aspect of the skull, evidencing the foramina of the medial and caudal walls of the orbit. Aso, Suborbital Arch; Cf, Set of foramina for the oculomotor (III), trochlear (IV), abducens (VI) and ophthalmic nerves, branch of the trigeminal nerve (V); F, Frontal Bone; Fo, Optic foramen; FSo, Supraorbital foramina; N, Nasal Bone; Oe, Scleral Ossicles; Prpo, Postorbital Process; Pz, Zygomatic Process; Zfc, Craniofacial Flexor Zone.

Near the dorsal margin of the orbit are the supraorbital foramina (Figure 7C). The rostral wall of the orbit (Figure 6) is formed by the lacrimal bone, which is fused with the frontal bone. A long orbital process starts from the lacrimal bone, which curves caudally, forming the suborbital arch, which completes the ventral margin of the orbit. The suborbital arch extends caudally until it crosses the zygomatic process, without fusion (Figure 7A). The free end of the postorbital process is located laterally to the free end of the zygomatic process. The zygomatic process is prominent and projects rostroventrally toward the suborbital arch. In the dissected cadaver, connective tissue was observed between the processes. The caudal margin of the orbit is prolonged with a short postorbital process directed ventrally. The medial wall of the orbit is formed by the interorbital septum, which is thick and presents the orbitocranial and interorbital fontanelles. The optic foramen is located ventral and caudal to the orbitocranial fontanelle and, laterally to this, there is a set of four small foramina (Figure 7D) for the passage of the oculomotor (III), trochlear (IV), abducens, and ophthalmic nerves, a branch of the trigeminal nerve (V).

## 4 Discussion

Ophthalmic abnormalities in birds can be classified as malformations, inflammation, degeneration, neoplasia, nutritional disorders, and traumatic injuries [17]. In psittacines, a retrospective study by Hvenegaard et al. [18] identified cataracts, uveitis, and ulcerative keratitis as the most common ophthalmic conditions. For birds under human care, most ocular diseases are linked to infectious processes, whereas in free‐living raptors, trauma is a more frequent cause, particularly affecting the posterior segment of the eye [18]. The incidence of ophthalmic lesions in ring‐necked parakeets has not been documented. However, establishing ophthalmic parameters for this species can aid in diagnosing eye conditions. Our study showed that ophthalmic examinations in ring‐necked parakeets can be performed under physical restraint without sedation, with tests completed in a few minutes. Since restraint can cause acute increases in corticosterone, leading to physiological stress responses, proper handling is essential to minimize these effects [19].

There are variations in the bony and muscular components of the skull of Psittaciformes, mainly in the suborbital arch and the pseudomasseter muscle, which are unique elements of this order and are not present in all species. Such variations are related to evolutionary and adaptive processes [20, 21]. Machado, Dos Santos, and Montiani‐Ferreira [22] listed species that fit into two classifications of the Psittaciformes orbit: open, which do not have the suborbital arch; and closed or complete, where there is a junction of the orbital and postorbital processes, forming the suborbital arch, and the ventral margin is completely bony. In ring‐necked parakeets, a long extension of the orbital process of the lacrimal bone is observed, which completes the orbit ventrally; however, this arch is not fused with the zygomatic process. The main difference between ring‐necked parakeets and birds with closed orbits is precisely the absence of a bony union between the suborbital arch and the zygomatic process, despite the fact that they cross and are very close. Evolutionary changes in parts of the skull of ring‐necked parakeets, with a high level of modularity, as demonstrated by Mitchell, Goswami, and Felice [23], may contribute to their adaptive success as an invasive species.

In parrots and macaws, there is a change in the color of the iris between adults and chicks, gradually varying from a brownish tone until assuming the color of the adult individual at approximately one year of age [24]. This same pattern of transition in the color of the eyes of juveniles was observed in ring‐necked parakeets; however, it begins in younger individuals, around 60 days of age. Further studies are needed to monitor the exact age at which this variation in iris color occurs, with the analysis of a larger number of individuals.

The EAPPTT for measuring the aqueous production of the tear film was first described for use in marmosets [25] and is currently a method with parameters described for use in several species, such as bats [26], guinea pigs [27], broad‐snouted caimans [28], ferrets [29], canaries, slider turtles, rats, and mice [30]. One of the advantages of this method is the use of a rigid tape with a small diameter (0.3 mm) for species that have a small eyeball [30], as in the case of the ring‐necked parakeet, whose eyeball diameter is 8.04 ± 0.49 mm. One of the limitations of the EAPPTT is the lack of standardization regarding the variety of endodontic paper point brands, which have different absorption capacities [31, 32]. Originally, the test was standardized using a specific size and brand of paper tip (Roeko Color‐Size 30, Langenau, Germany) [25]. However, this brand was commercially unavailable in Brazil, and in the development of this study, it was decided to use another manufacturer (Dentsply Color size 30) that has also been used in rabbits [33]. Thus, the results found in this study refer to standardized measurements with this brand of paper tip, and the value found for the EAPPTT was 7.57 ± 1.3 mm/min. The value found is lower than that of other psittacine species, such as the blue‐and‐yellow macaw (16.74 ± 1.38 mm/min), Lear's macaw (15.37 ± 1.22) [8], *Amazona aestiva* (13.1 ± 1.4 mm/min) and *Amazona amazonica* (14.9 ± 1.6 mm/min) [10], but close to cockatiels (8.7 mm/min) [12] and caatinga parakeets (8.74 ± 2.0 mm/min) [14], which are animals of similar size to ring‐necked parakeets. However, the studies cited were carried out with different brands of paper points. The material was easily inserted into the conjunctival fornix, and the animals did not appear to be uncomfortable.

Knowledge of the corneoconjunctival microbiota of different bird species is important for the adequate evaluation of patients facing an infectious process. Most of the bacterial isolates in this study were gram‐positive bacteria, as found in other investigations in birds [7, 8, 14, 34, 35, 36, 37]. Bacteria of the genus *Staphylococcus* were the most frequently isolated, corroborating studies in owls [34, 38] and macaws [8], but different from flamingos, where a predominance of the genus *Enterococcus* was observed [39], and bustards with a predominance of *Micrococcus* [40]. Bacteria of the genus *Staphylococcus* are part of the resident flora of birds and inhabit the external surface of the cornea, with the function of ocular protection, although they can become pathogenic under imbalances in the ocular microbiota [35]. This may occur due to the indiscriminate use of antibiotics or changes in the immune system, which may lead to excessive growth of gram‐negative bacteria and fungi [35], predisposing the eye to infections. The main causes of infectious conjunctivitis in psittacines are of bacterial origin, with the most common isolates being *Chlamydophila psittaci* , *Mycoplasma* sp., *Streptococcus* sp., and *Pseudomonas aeruginosa* [6]. New studies are needed to determine the pathogenicity of the agents found, in addition to the antibiogram exam, to assess the resistance and sensitivity profile to antibiotics used in routine clinical practice in birds.

In birds with small ocular diameters, such as ring‐necked parakeets, IOP can be measured using a rebound tonometer. The procedure was well tolerated by these birds, without the need for sedation or application of anesthetic eye drops. In the study by Karimi et al. [15] with eight ring‐necked parakeets, the mean IOP was 6.25 ± 1.75 mmHg, with a statistical difference in the values found between the right and left eyes. The study was performed with a Tonovet rebound tonometer (Jorgensen Laboratories, Loveland, CO, USA) in the “P” calibration mode [15]. The value is different from that found in the present study, of 12.47 ± 1.51 mmHg, in the evaluation of 66 eyes with Tonovet Plus (Icare Finland Oy, Helsinki, Finland) in the “D” calibration mode. The differences can be explained by the use of another tonometer manufacturer and calibration modes, which highlights the lack of standardization in the measurement of IOP in birds. The value found is similar to references for blue‐and‐yellow macaws (11.49 ± 0.22 mmHg) [8], red‐headed vultures (11.7 ± 1 mmHg) [41], and pigeons (11.7 ± 1.6 mmHg) [42]. However, it is higher than that found in Lear's macaws (7.0 ± 2 mmHg) [8] and lower than that in penguins (29.1 ± 7.16 mmHg) [5]. There is variability in IOP values in different bird species due to anatomical differences, such as the curvature and thickness of the cornea and the size of the eyeball [43].

Ring‐necked parakeets have a CTT value of 2.46 ± 0.5 cm, which is higher than that of Lear's macaws (1.5 ± 0.5 cm) [8], striped owls (0.81 ± 0.89 cm) [34], and blue‐fronted parrots (1.0 cm) [9], and similar to that found in caracaras (2.46 ± 1.1 cm) [37]. Higher esthesiometry values are inversely proportional to sensitivity; that is, the higher the value, the lower the corneal sensitivity threshold. Factors such as age may influence this parameter, with reports of some young raptor species having greater corneal sensitivity than adults [44]. However, this factor was not tested in ring‐necked parakeets.

Central corneal thickness measurements were performed using an ultrasonic pachymeter. Currently, it is the most accurate method for measuring this parameter in vivo and with the animal conscious. The instrument works by measuring the time required for ultrasonic energy to pass through the cornea and converts this measurement into a thickness value [45]. The average central corneal thickness in the species is 0.12 mm. This value is lower than that found in caracaras (0.31 mm ± 0.02) [37], puffins (0.24 mm) [46], and chickens (0.24 mm) [45]. Between males and females, it was the only parameter evaluated in which there was a statistically significant difference (*p* = 0.2), with the cornea being thicker in females than in males, and in other birds such as the striped owl, the opposite was reported [34] that the cornea of males has a greater thickness value.

Ultrasonography allows the measurement and evaluation of internal ocular structures as a tool for diagnostic aid in birds with ophthalmic alterations [47]. The B‐mode ocular ultrasound examination was well tolerated by the birds, using only anesthetic eye drops and ultrasound gel, which left the feathers in the periocular region moist. The results obtained are similar to those found in other psittacines, with variations consistent with the size of the different species. Ocular biometry has general values lower than those found in parrots [10] and macaws [8] and higher than in parakeets [13]. In comparison with the striped owl, the axial length of their ocular bulb [48] is more than twice the length observed in ring‐necked parakeets, since owls have nocturnal habits and therefore their eyes are larger to allow greater light input [49]. The ocular pecten can be effectively visualized and measured using B‐mode ultrasound, which is considered the most reliable imaging method for diagnosing this structure, surpassing both computed tomography and magnetic resonance imaging [50]. It could also be evaluated using Doppler ultrasound imaging, as in studies with harpies, demonstrating its circulatory activity [51].

## 5 Final Considerations

The data obtained in this research enhances the ability to diagnose ocular conditions in ring‐necked parakeets, a species commonly kept as pets. The study offers a more detailed understanding of the anatomical characteristics of the orbit and external ocular features. The establishment of specific ophthalmic parameters, unprecedented in this species, will provide veterinarians with valuable reference points for diagnosing and managing eye‐related issues. Additionally, this study underscores the need for further complementary exams, such as investigations into the prevalence of ocular diseases, ocular histology, and assessments of visual acuity and color distinction. These future studies will deepen our understanding of ring‐necked parakeets' eye health and their visual biology.

## Author Contributions

**Fernanda Taques Wendt:** conceptualization, investigation, methodology, supervision, validation, writing – original draft. **Fabiano Montiani‐Ferreira:** project administration, supervision, validation, writing – review and editing. **Cecília Capacchi Dall’agnol:** resources. **Thiago Francisco Costa Solak:** resources. **Franz Riegler Mello:** resources. **Breno Castello‐Branco Beirão:** investigation, resources, supervision, writing – review and editing. **Marcello Machado:** methodology, supervision, validation, writing – review and editing. **Rogério Ribas Lange:** project administration, resources, supervision, validation, writing – review and editing.

## Ethics Statement

This study complies with the ARVO Statement for the Use of Animals in Ophthalmic and Vision Research and was approved by the Animal Ethics Committee of the Federal University of Paraná. Animals were not captured, restrained, sedated, or anesthetized solely for the purposes of this study.

## Conflicts of Interest

The authors declare no conflicts of interest.
